# Results of Pulmonary Valve Replacement with The INSPIRIS RESILIA Prosthesis: A Systematic Review

**DOI:** 10.21470/1678-9741-2025-0441

**Published:** 2026-09-08

**Authors:** Carlos J Pérez Rivera, Guillermo Portilla-Rodríguez, Leandro Guarín-Muñoz, Diego Penagos-López, Oscar Sánchez-Colmenares, William Rios, Juan Parra, Federico Núñez-Ricardo

**Affiliations:** 1 Department of Cardiothoracic Surgery, School of Medicine, Universidad de El Bosque, Bogotá, Colombia.; 2 Division of Cardiovascular Surgery, Department of Surgery, Fundación Clínica Shaio, Bogotá, Colombia.; 3 Division of Cardiothoracic Surgery, Department of Surgery, Fundación Santa Fe de Bogotá, Bogotá, Colombia.

**Keywords:** INSPIRIS RESILIA, Pulmonary Valve, Congenital Heart Disease, Valve Replacement, Bioprosthesis

## Abstract

**Introduction:**

Pulmonary valve replacement is increasingly common in patients with congenital heart disease. The limited durability of conventional bioprostheses has led to the introduction of the INSPIRIS RESILIA valve, initially designed for the aortic position. However, evidence on its performance in the pulmonary position is limited and heterogeneous.

**Methods:**

A systematic review was conducted in accordance with the Preferred Reporting Items for Systematic Reviews and Meta-Analyses statement in PubMed®, Excerpta Medica dataBASE, Cochrane Library, and ClinicalTrials. Observational studies with patients undergoing pulmonary valve replacement with the INSPIRIS RESILIA bioprosthesis were included. Data on baseline characteristics, transvalvular gradients, mortality, complications, prosthetic insuficiency, and need for reintervention were extracted. This study was registered in the International Prospective Register of Systematic Reviews (CRD420251117859).

**Results:**

Three retrospective cohort studies and two retrospective case series were identified that included pediatric and adult populations, mainly with a diagnosis of tetralogy of Fallot. The findings were consistent in showing adequate perioperative safety. Smaller studies showed favorable results. In contrast, larger studies with longer follow-up reported more rapid progression to moderate/severe pulmonary insuficiency and shorter durability of the bioprosthesis.

**Conclusion:**

INSPIRIS RESILIA may be associated with acceptable short-term outcomes in pulmonary valve replacement, but its medium- and long-term durability may be inferior to that of reference bioprostheses. Medium- and long-term safety remains inconclusive due to limited follow-up, modest sample sizes, heterogeneity in definitions, possible confounding by anatomical substrate, and study limitations.

## INTRODUCTION

Pulmonary valve replacement (PVR) has been on the rise, with an increase from 0.4 to 0.6 replacements per million inhabitants from 1976 to 2021, with congenital heart disease (CHD) being the main indication^[1,2]^. These affect nine out of every 100 live births, and 20% of them have malformations of the pulmonary valve or the right ventricular outfow tract (RVOT)^[3]^. According to Mckenzie et al.^[4]^, the most common CHDs are tetralogy of Fallot (TOF) and pulmonary atresia. Currently, the early surgical mortality rate for CHD repair is 0 to 2%, meaning that the vast majority of patients reach adulthood, and half of the patients who survive surgical repair of TOF will require PVR at age 30.

To meet this growing need for PVR, there are different options such as homografts and bovine jugular vein conduits, which have limitations in terms of availability and size variety, and mechanical and biological prosthetic valves, which are associated with higher rates of valve thrombosis^[5]^. On the other hand, the aortic and pulmonary valves have similar mechanical, structural, and architectural characteristics, which is why biological aortic valve prostheses are widely used in the pulmonary position, the most commonly used being the Carpentier-Edwards PERIMOUNT bovine pericardium valve with stent^[5,6]^.

The longevity of biological valve prostheses is inherently limited; although durability may vary depending on the patient and valve design, structural degeneration and hemodynamic dysfunction are inevitable processes that, over time, require reintervention^[7,8]^. Faced with this challenge, the INSPIRIS valve, made from bovine pericardium and featuring RESILIA technology, emerged. It initially demonstrated excellent results in aortic valve replacement, reducing structural degeneration and prolonging durability^[9,10,11]^. Despite the expansion of the use of the INSPIRIS valve to the pulmonary position, evidence supporting its performance in this context is scarce and the results are inconclusive. To address this uncer tainty, the purpose of this study is to evaluate the effectiveness and safety of the INSPIRIS RESILIA biological valve in PVR.

## METHODS

### Design

This systematic review was conducted following the Preferred Reporting Items for Systematic Reviews and Meta-Analyses (or PRISMA) guidelines^[12]^. In addition, it was previously registered in the International Prospective Register of Systematic Reviews (or PROSPERO) (registration number CRD420251117859).

A systematic search was conducted in the MEDLINE/PubMed®, Cochrane Library, Excerpta Medica dataBASE (or EMBASE), and ClinicalTrials databases. Controlled terms and free words related to “Inspiris Resilia”, “Inspiris”, “Edwards Inspiris Resilia”, “pulmonary valve replacement”, and “right ventricular outfow tract” were used. No language or publication date restrictions were applied.

### Selection Criteria

Observational studies, clinical trials, and case series with at least fve patients undergoing PVR with INSPIRIS RESILIA and reporting clinical or hemodynamic outcomes were included. Isolated case reports, narrative reviews, editorials, letters to the editor, conference abstracts, and studies in which the INSPIRIS valve was used exclusively in the aortic position were excluded.

### Data Collection Process

Two independent reviewers screened titles and abstracts to identify potentially eligible studies (LGM and GP). Full-text articles were subsequently reviewed to confirm eligibility, and any discrepancies were resolved by consensus. Study selection and data extraction were performed manually, without the use of dedicated screening software.

Data extraction was independently conducted by both reviewers using a standardized table. Extracted datasets were compared, and no discrepancies were identified.

Collected variables included population characteristics, prior interventions, surgical technique, transvalvular gradients, early and late mortality, morbidity, prosthetic insuficiency, stenosis, need for reintervention, and duration of follow-up.

### Outcome Definitions and Data Handling

At the protocol design stage, we planned to adopt standardized outcome definitions based on the Valve Academic Research Consortium-3 (VARC-3) consensus to define prosthetic valve dysfunction and prosthetic valve failure.

According to VARC-3, structural valve deterioration is defined as intrinsic prosthetic valve dysfunction resulting in moderate or greater prosthetic regurgitation and/or hemodynamically relevant stenosis, whereas prosthetic valve failure is defined by the presence of severe prosthetic regurgitation or severe prosthetic stenosis, valve-related death, or the occurrence of valve reintervention^[13]^. In VARC-3, valve reintervention is defined as any repeat surgical or transcatheter procedure performed to repair, modify, or replace a previously implanted prosthetic valve, including valve replacement, valve-in-valve procedures, or other interventions undertaken to treat prosthetic valve dysfunction^[13]^.

However, during data extraction it became evident that the included studies employed heterogeneous methodologies and frequently lacked explicit or suficiently detailed criteria for adjudicating valve-related outcomes. Consequently, uniform application of VARC-3 definitions across all studies was not methodologically appropriate. Therefore, valve-related outcomes were extracted and reported as defined by each individual study, preserving the original methodological framework of the included reports.

### Data Synthesis and Statistical Analysis

The included studies showed relevant clinical and methodological heterogeneity. Differences were observed regarding implantation in the native RVOT *vs.* valved conduits, inclusion of pediatric and adult populations, variability in surgical techniques, non-uniform definitions of outcomes, and heterogeneous follow-up durations. In addition, the available studies included relatively small sample sizes, and the study populations were highly heterogeneous. Taken together, these factors limited the feasibility of performing a meaningful quantitative synthesis or subgroup analyses, as pooling the results could have led to misleading conclusions. Therefore, the findings of this review were summarized using a narrative synthesis approach.

### Methodological Quality and Risk of Bias Assessment

The quality of the included studies was assessed using the Newcastle-Ottawa Scale for observational studies and the Joanna Briggs Institute checklist for case series^[14,15]^.

The risk of bias in the included studies was assessed using the Risk Of Bias In Non-randomized Studies of Interventions, version 2 (ROBINS-I V2) tool. This tool allows for a structured assessment of the risk of bias in observational studies that estimate the effects of interventions, comparing them conceptually with an ideal randomized clinical trial (target trial). The assessment was performed at the outcome level, considering the six domains of the tool. For each domain, a judgment of low, moderate, serious, or critical risk of bias was assigned, according to the instrument's guidelines. Discrepancies in judgments were resolved by methodological consensus.

## RESULTS

Three retrospective observational studies and two case series were identified that evaluated the clinical and hemodynamic performance of the INSPIRIS RESILIA bioprosthesis in the pulmonary position (Figure 1). Collectively, these studies included a total of 204 patients, of whom 53.9% (110) were male, encompassing both pediatric and adult populations with CHD, predominantly TOF, and reported early and medium-term outcomes.

**Fig. 1:**
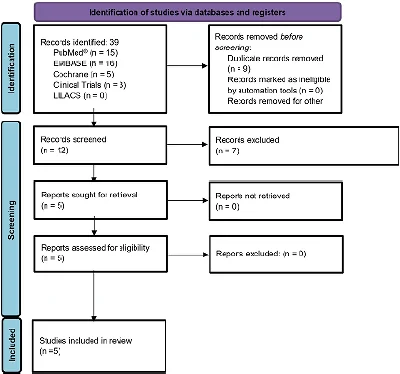
Flow diagram of studies assessed for eligibility.

### Quality of Evidence

The assessment of methodological quality, performed using the Newcastle-Ottawa Scale and the Joanna Briggs Institute checklist, showed that the included studies were generally of high quality, with scores between 4 and 6 out of a possible 8 (Table 1) and adequate compliance with most of the items evaluated. Although some limitations were observed in the comparability and specific criteria of certain studies, overall, the studies showed consistency in population selection, robustness in the reporting of results, and an acceptable methodological level for the synthesis of evidence.

**Table 1:** Methodological quality and risk of bias assessment of the included studies (NOS, JBI, and ROBINS-I).

Methodological quality					
Author	NOS criteria				
	Selection	Comparability (0 - 1 )	Results	Total	Overall quality
	(0-4)		(0-3)	(0-8)	
Ragheb DK^[8]^	***	*	**	**6/8**	High
Sengupta A^[17]^	**	0	* *	**4/8**	High
Nguyen SN^[18]^	**	*	* **	**6/8**	High
Author	JBI criteria									
	1	2	3	4	5	6	7	8	9	10
Said SM^[16]^	Yes	Yes	Yes	Yes	Yes	Yes	Yes	Yes	Yes	Yes
Arribas-Leal JM^[19]^	Yes	Yes	Yes	Yes	Yes	Yes	Yes	Yes	Yes	Yes
Author	Risk of bias						
	ROBINS-I						
	Domain 1	Domain 2	Domain 3	Domain 4	Domain 5	Domain 6	Overall risk
Ragheb DK^[8]^	Moderate	Low	Moderate	Low	Low	Moderate	Moderate
Sengupta A^[17]^	Moderate	Low	Moderate	Low	Low	Moderate	Moderate
Nguyen SN^[18]^	Moderate	Low	Moderate	Moderate	Low	Moderate	Moderate

JBI=Joanna Briggs Institute; NOS=Newcastle-Ottawa Scale; ROBINS-l=Risk Of Bias In Non-randomized Studies of Interventions

### Risk of Bias

The risk of bias assessment was performed only for comparative observational studies, using the ROBINS-I V2 tool. Non-comparative studies (case series) were not included in this assessment, as their design does not allow for causal inference or comparative estimation of effects. In the studies evaluated, the overall risk of bias was moderate, mainly determined by residual confounding and possible biases in participant selection, while the domains of intervention classification, missing data, and outcome measurement presented, in general, a low risk of bias.

Table 2 presents the sociodemographic characteristics of participants in the fve included studies, conducted in the United States of America and Spain. A total of 204 patients were included. The weighted average age was 20.1 years, and 53.9% of the participants were male. The populations demonstrated relative homogeneity in baseline diagnoses, with TOF predominating across cohorts, despite substantial heterogeneity in age distribution, prior surgical history, implantation substrate, and follow-up duration. Studies show a high prevalence of previous cardiac interventions, including repair of TOF and pulmonary valvuloplasty. Other CHDs, such as pulmonary stenosis and truncus arteriosus, were less frequently represented, reflecting a focus on patients with complex congenital pathology and a clinical course characterized by multiple prior procedures.

**Table 2.** Characterization of the study population.

Primary author	Country	Patients (n)	Age (years)	Sex	Previous intervention n (%)	Most frequent diagnosis n (%)
Said SM^[16]^	United States of America	27	22.26 ± 14.99	Male: 12	TOF repair with or without PA: 15 (55.6%)	TOF/PS: 13 (48.1%)
				Female: 15	Transcatheter balloon pulmonary valvuloplasty/ PV perforation: 5 (18.5%)	Congenital PS: 6 (22.2%)
					Systemic artery to pulmonary artery shunt: 4 (14.8%)	TOF/PA: 2 (7.4%)
					Pulmonary artery branch stenting: 3 (11.1%)	PA/IVS: 2 (7.4%).
					Open pulmonary valvulotomy: 2 (7.4%).	TOF/pulmonary valve absence: 1 (3.7%)
						Truncus arterio-sus: 1 (3.7%)
						TOF/atrial septal defect: 1 (3.7%)
						Previous Ross procedure: 1 (3.7%)
Ragheb DK^[8]^	United States of America	62	13.8 (6.7 - 25.1)	Male: 32	Repair without conduit or valve: 46 (74%)	TOF: 39 (63%)
				Female: 30	Repair with conduit: 9 (15%)	Pulmonary valve stenosis: 9 (15%)
					Repair with bioprosthetic valve: 3 (5%)	PA/IVS: 5 (8%)
					Pulmonary balloon valvu-loplasty: 2 (3%)	Others: 9 (15%)
					None: 2 (3%)	
Sengupta A^[17]^	United States of America	24	26 (4 - 60)	Male: 10	TOF repair: 24 (100%)	TOF: 22 (91.7%)
				Female: 14	Transcatheter pulmonary valve procedures: 6 (25%)	DORV/TOF: 2 (8.3%)
Nguyen SN^[18]^	United States of America	70	19.5 (12.9 - 31.6)	Male: 42	TOF repair: 34 (48.6%)	TOF/PS: 30 (42.9)
				Female: 28	Ross procedure: 7 (10%)	TOF/PA: 10 (14.3)
					RV-PA duct replacement: 14 (20%)	Truncus arterio-sus: 5 (7.1%)
					Systemic-pulmonary shunt: 10 (14.3%)	Congenital PS: 3 (4.3%)
					Surgical PVR: 5 (7.1%)	DORV/TOF: 1 (1.4%)
					Truncus arteriosus repair: 6 (8.6%)	PA/IVS: 3 (4.3%)
					Pulmonary valvulotomy: 3 (4.3%)	Others: 8 (7.1%)
					Arterial switch operation: 2 (2.9%)	
					Rastelli operation: 2 (2.9%)	
Arribas-Leal JM^[19]^	Spain	21	31 ± 16.6	Male: 14	Previous intervention: 18 (85.7%)	TOF: 14 (66.6%)
				Female: 7	Not specified	

DORV=double-outlet right ventricle; IVS=intact ventricular septum; PA=pulmonary atresia; PS=pulmonary stenosis; RV=right ventricle; TOF=tetralogy of Fallot; PV=pulmonary valve; PVR=pulmonary valve replacement

### Indications for Pulmonary Valve Replacement

In the study conducted by Said et al.^[16]^, the most frequent indications for PVR were related to the presence of symptoms. Dyspnea was the predominant clinical finding (nine patients; 33.3%), followed by exercise intolerance (seven patients; 25.9%), fatigue (fve patients; 18.5%), and palpitations (one patient; 3.7%). Other indications included objective findings such as significant right ventricular dilation (end-diastolic volume index ≥ 150 ml/m^2^) assessed by cardiac magnetic resonance imaging, elevated right ventricular pressure secondary to obstruction of the RVOT, need for aortic root replacement in patients with prior Ross procedure, and recurrent pulmonary duct endocarditis (one case with persistent bacteremia requiring reoperation)^[16]^. In Ragheb DK's study, pulmonary insuficiency was present in 43 (69%), pulmonary stenosis in seven (11%), and both conditions in 12 (18%) patients. The most frequent diagnoses were TOF in 39 patients (63%), pulmonary valve stenosis in nine (15%), pulmonary atresia with intact ventricular septum in five, and other unspecified conditions in nine patients.

In Sengupta's study, there was symptomatic severe pulmonary insuficiency with progressive right ventricular dilatation in 19 patients (79.2%); severe pulmonary and tricuspid regurgitation with right ventricular dysfunction in two (8.3%); severe RVOT obstruction or pulmonary stenosis in two (8.3%); and Melody™ valve endocarditis in one (4.2%)^[17]^. In the study by Nguyen SN et al.^[18]^, the indications for PVR were pulmonary insuficiency in 41 patients (58.6%), pulmonary insuficiency and stenosis in 19 (27.1%), and pulmonary stenosis in 12 (17.1%). Finally, the study led by Arribas-Leal et al.^[19]^ did not record this information.

### Primary Outcome

Table 3 describes the primary and secondary outcomes associated with the follow-up period. In the study by Sengupta A et al.^[17]^, no reoperations were performed; however, they reported that one patient required re-exploration due to bleeding (4.2%), another developed a deep sternal wound infection that required surgical debridement (4.2%), one patient required readmission for heart failure (4.2%), and another underwent planned implantation of an automatic defbrillator (4.2%). At one year, all patients had, at most, mild pulmonary insuficiency or stenosis. The severity of stenosis did not change from discharge to one year follow-up.

**Table 3.** Primary and secondary results.

Primary author	Follow-up (months)	Early or late mortality (number of patients)	Morbidity	Reinterventions	Insufficiency (number of patients)	Time of onset of PR	Progression of PR (number of patients)	Free of prosthetic failure	Transvalvular gradient (mmHg)
Said SM^[16]^	16 ± 8	0	Calcified pulmonary duct, residual major aortopulmonary collateral arteries, and bilateral stenosis of the pulmonary arterial branches: 1	Unifocalization of residual major aortopulmonary collateral arteries, bilateral pulmonary arterioplasties, and conduit replacement in the perioperative period: 1	13 patients (48%):	At 8 months	Severe: 3 (11 %)	Not reported.	Average maximum gradient: 13.8 ±5.7
			AF: 2	Transcatheter valve implantation: 3; 1 at 9 months and 2 at 29 months from PVR	Mild PR: 7				
					Moderate PR: 6				
Ragheb DK^[8]^	19 (25^th^ - 75^th^ %, 13-31; maximum 42)	0	Not reported.	Surgical PVR due to endocarditis with thrombus and negative culture at 8 months: 1	Moderate to severe PR: 9 (5 in the 2-year group and all in the 3-year group)	At 2 years: 12 ±5%	Not reported.	Not reported.	Average gradient: 5 (4-7)
				PVR due to thrombosis with associated hemodynamic collapse, secondary to severe PR and RVOT obstruction at 13 months: 1		At 3 years: 31% ±9%			
Sengupta A^[17]^	29 (IQR: 16-30; 12-36)	0	0	0	Mild PR at discharge: 2.4 (10%)	Not reported.	(7.2) 30%	Not reported.	Average maximum gradients at discharge: 7.4 ±2.3;
					Mild PR at one month: 2.6 (11%)				at 30 days: 5.8 ±3.1;
					Mild PR at one year: 8.4 (35%)				at one year: 6.3 ±3.5.
Nguyen SN^[18]^	26.6 (IQR, 12.4-41.1)	Late mortality due to cardiogenic shock: 1	0	Severe proximal and distal narrowing of the valved conduit with moderate PR. Balloon angioplasty was performed and a stent was implanted at 21.8 months; the proximal stent was placed across the valve, and 16.2 months later, a 23 mm SAPIEN3 Ultra valve was implanted: 1 patient.	The presence of at least moderate PR:	10.2 months (IQR: 5.8 -14.9)	Not reported.	At 6 months: 91.1 ±3.9%	Not reported.
				Severe mixed PR/PS of the valve implanted in the native RVOT, requiring percutaneous implantation of an 18 mm Melody™ valve (Medtronic) 12.2 months later: 1 patient.	At 6 months: 4.0 ± 2.8%			At 12 months: 73.4 ± 6.8%	
					At 12 months: 19.1 ±6.1%			At 24 months: 53.5 ± 9.3%	
					At 24 months: 45.8 ± 9.6%				
					Moderate or greater PR: 21 patients (70%).				
					PR + PS (mixed): 5 patients (16.7%).				
					Isolated PS: 3 patients (10%).				
Arribas-Leal, j_M_[19]	31 ±16.6	Early mortality due to multiple organ failure: 1	0	0	0	Not reported.	Not reported.	Not reported.	Maximum average gradient: 17 ±7.23.

AF=atrial fibrillation; IQR=interquartile range; PVR=pulmonary valve replacement; PR=pulmonary regurgitation; PS=pulmonary stenosis; RVOT=right ventricular outflow tract

In the study by Nguyen SN et al.^[18]^, seven complications were documented: two unplanned reoperations unrelated to the valve, one surgical site infection or mediastinitis, one case of prolonged mechanical ventilation, one cardiac arrest, and one patient who required permanent pacemaker implantation; an additional patient required extracorporeal membrane oxygenation support. The absence of valve failure (defined as at least moderate valve regurgitation and/or stenosis, or any reintervention on the valve or pulmonary conduit) at six, 12, and 24 months was initially lower in the INSPIRIS group; however, as time passed, valve failure increased compared to the control group (91.1% ± 3.9%, 73.4% ± 6.8%, and 53.5% ± 9.3%, respectively) compared to the non-INSPIRIS group (PERIMOUNT, Contegra™, homografts, Mosaic™, and Autus) (94.1% ± 2.9%, 90.2% ± 3.9%, and 78.5% ± 5.9%). In addition, two patients (1.4%) required heart transplantation due to end-stage heart failure. The study by Arribas-Leal et al.^[19]^ reported no postoperative complications, such as late mortality or progression of prosthetic failure, nor any need for reoperations^[19]^.

### Secondary Outcome

Given the shorter durability of the INSPIRIS valve in native RVOT, Nguyen et al.^[18]^ investigated its performance compared to the PERIMOUNT valve. The absence of valve failure at six, 12, and 18 months was similar between the two groups (100.0% ± 0.0%, 90.9% ± 6.1%, 73.7% ± 10.3% for INSPIRIS *vs.* 94.7% ± 3.6%, 91.6% ± 4.7% for PERIMOUNT). However, the absence of at least moderate pulmonary regurgitation at six, 12, and 18 months was lower in the INSPIRIS valve from the outset and continued to deteriorate with prolonged follow-up (100.0% ± 0.0%, 95.5% ± 4.4%, 77.4% ± 10.1%) compared with the PERIMOUNT group (97.4% ± 2.5%, 97.4% ± 2.5%, 97.4% ± 2.5%).

### Heterogeneity Across Included Studies

Clinical and methodological heterogeneity was observed across the included studies. Differences were noted in patient age (pediatric *vs.* adult populations), surgical approach and implantation substrate (native RVOT *vs.* valved conduits), valve sizes, outcome definitions, and duration of follow-up, which ranged from 12 to 41 months. This heterogeneity precluded meaningful quantitative pooling of results or formal subgroup analyses.

## DISCUSSION

Overall, the included studies showed consistent results regarding the early safety of the INSPIRIS RESILIA bioprosthesis. Series such as that by Arribas-Leal et al.^[19]^ reported no early mortality or major complications, findings consistent with those of Sengupta et al.^[17]^, who also described low and stable transvalvular gradients in the frst year of follow-up.

This evidence supports the adequate initial tolerance of the prosthesis in different surgical and population settings. However, studies with larger sample sizes and longer follow-up periods, such as those by Ragheb et al.^[8]^ and Nguyen et al.^[18]^, identified a higher incidence of moderate or severe pulmonary insuficiency in the medium term in patients with INSPIRIS, compared with reference bioprostheses such as Mosaic™ and PERIMOUNT. This pattern was more evident when the valve was implanted in the native RVOT, where durability was limited compared to implantation in valved conduits^[8,18]^. These findings suggest that the anatomical and hemodynamic characteristics of the native RVOT may infuence the performance of the prosthesis. Thus, the available literature suggests that INSPIRIS RESILIA may be associated with acceptable short-term safety; however, concerns remain regarding its medium- and long-term durability, as the evidence is derived from retrospective series with predominantly ≤ 36 months of follow-up, modest sample sizes, heterogeneity in definitions and measurement timing, limited power for rare events, and potential confounding by anatomical substrate (native RVOT *vs.* conduits).

In this regard, recent literature has suggested that the dynamics of the pulmonary root could pose an additional challenge for the INSPIRIS valve. Unlike the aortic annulus, which is frmly supported by the fbrous skeleton of the heart, the pulmonary root is a highly distensible structure with greater anatomical variability. This elastic behavior, coupled with the absence of a rigid annulus, could generate radial forces that compromise valve coaptation, favoring the development of central prosthetic insuficiency despite preserved cusp mobility^[18]^. Hence, the hypothesis of partial incompatibility between the valve design (conceived for the aortic root) and the physiology of the right side of the heart is plausible and deserves validation in experimental biomechanical studies.

On the other hand, the findings of less regurgitation in patients who received an INSPIRIS valved conduit suggest that the external tubular support could play a protective role in the durability of the prosthesis. The relative rigidity of a vascular graft contrasts with the dynamic expansion of the pulmonary root, offering a more stable geometry that would favor valve coaptation and reduce the risk of early incompetence^[18]^.

This opens the door to considering the systematic inclusion of the valve in tubular grafts when used orthotopically, without limiting the possibility of future valve-in-valve procedures. However, this strategy requires further clinical validation, given that current experience is limited and most multicenter trials have excluded patients with valved conduits.

In this broader context of PVR durability, comparative studies evaluating alternative prosthetic strategies provide additional perspective.

Two retrospective studies by Cocomello et al.^[20]^ and Batlivala et al.^[21]^ evaluated bioprosthetic valves and valved homografts using comparable clinical and echocardiographic variables. Both studies reported acceptable early and mid-term outcomes, supporting the short-term safety of contemporary bioprosthetic PVR strategies. Differences in durability were observed across studies. Cocomello et al.^[20]^ reported lower reintervention rates with pulmonary homografts, whereas Batlivala et al.^[21]^ did not identify significant differences, likely reflecting heterogeneity in conduit type and patient characteristics.

The main strength of this review is that it constitutes the most comprehensive synthesis of published evidence on the INSPIRIS RESILIA valve in the pulmonary position, integrating experiences from different continents and both pediatric and adult populations. In addition, the analysis included comparisons with other bioprostheses, allowing the relative performance of the valve to be contextualized.

In clinical terms, the findings suggest that the INSPIRIS RESILIA valve may represent a safe option for patients with an indication for short-term pulmonary replacement, especially in scenarios where the availability of homogeneous conduits is limited.

However, the higher incidence of prosthetic failure reported in some studies indicates that its use should be carefully evaluated in young patients or those expected to undergo multiple future reoperations. Scientifically, these results highlight the need to reassess whether the advantages observed in the aortic position can be extrapolated to the low-pressure circulation of the right side of the heart.

Future prospective studies with larger sample sizes and longer follow-up periods are essential to clarify the true durability of the bioprosthesis in the pulmonary position and its performance. The development of multicenter registries and direct comparisons with reference valves such as Mosaic™ or PERIMOUNT could provide robust evidence. Likewise, research on the interaction of RESILIA technology with the hemodynamic characteristics of the right ventricle could guide design modifications and optimize long-term results.

### Limitations

All included studies were retrospective and exhibited substantial heterogeneity in surgical techniques, valve sizes, implantation substrate, and patient populations, which limited the ability to draw robust inferences. Follow-up duration was relatively short, with limited data beyond three years. In addition, several studies did not systematically report clinically relevant outcomes such as late mortality, need for reintervention, or progression of prosthetic insuficiency. Furthermore, valve-related outcomes were defined and adjudicated inconsistently across studies, with frequent absence of standardized or composite endpoint definitions, which precluded direct quantitative comparison and limited the synthesis of valve dysfunction and failure rates to a descriptive analysis based on study-specific definitions.

### Takeaway

* INSPIRIS RESILIA demonstrates good short-term safety but potentially reduced long-term durability compared with standard bioprostheses, especially in the native right ventricular outfow tract.
* Implantation within valved conduits appears to improve prosthetic stability and reduce regurgitation.
* Limited, heterogeneous evidence highlights the need for larger, prospective, long-term studies to better define INSPIRIS RESILIA’s performance and durability.

## CONCLUSION

This systematic review shows that the INSPIRIS RESILIA bioprosthesis offers an adequate safety profle in the perioperative period, with low early mortality and favorable transvalvular gradients. However, medium- and long-term follow-up findings suggest a trend toward higher incidence of prosthetic failure and lower durability, especially when the valve is implanted in the native RVOT but remains inconclusive because the available evidence comes from retrospective series with predominantly ≤ 36 months of follow-up, modest sample sizes, heterogeneity in definitions and measurement times, low power for rare events, and possible confounding by anatomical substrate (native RVOT *vs.* conduits).

## Data Availability

The authors declare that the data supporting the findings of this study are available within the article.
